# Phospholipase D1 promotes cervical cancer progression by activating the RAS pathway

**DOI:** 10.1111/jcmm.17439

**Published:** 2022-06-30

**Authors:** Meiying Song, Qianlong Meng, Xuan Jiang, Jun Liu, Meizhu Xiao, Zhenyu Zhang, Jing Wang, Huimin Bai

**Affiliations:** ^1^ Department of Obstetrics and Gynecology, Beijing Chao‐yang Hospital Capital Medical University Beijing China; ^2^ Department of Obstetrics and Gynecology, Fuxing Hospital Capital Medical University Beijing China; ^3^ Department of Diagnostics of Clinical Laboratory, Beijing Chao‐yang Hospital Capital Medical University Beijing China; ^4^ The Clinical Research Center, Beijing Chaoyang Hospital Capital Medical University Beijing China

**Keywords:** biological characteristics, cervical cancer, CRISPR/Cas9 system, PLD1, RAS pathway

## Abstract

This study aimed to further investigate the effect of *PLD1* on the biological characteristics of human cervical cancer (CC) cell line, CASKI and the potential related molecular mechanism. CRISPR/Cas9 genome editing technology was used to knock out the *PLD1* gene in CASKI cells. Cell function assays were performed to evaluate the effect of *PLD1* on the biological function of CASKI cells in vivo and in vitro. A *PLD1*‐overexpression rescue experiment in these knockout cells was performed to further confirm its function. Two PLD1‐knockout CASKI cell lines (named PC‐11 and PC‐40, which carried the ins1/del4 mutation and del1/del2/ins1 mutation, respectively), were constructed by CRISPR/Cas9. PLD1 was overexpressed in these knockout cells (named PC11‐PLD1 and PC40‐PLD1 cells), which rescued the expression of PLD1 by approximately 71.33% and 74.54%, respectively. In vivo, the cell function assay results revealed that compared with wild‐type (WT)‐CASKI cells, the ability of PC‐11 and PC‐40 cells to proliferate, invade and migrate was significantly inhibited. The expression of H‐Ras and phosphorylation of Erk1/2 (p‐Erk1/2) was decreased in PC‐11 and PC‐40 cells compared with WT‐CASKI cells. PC‐11 and PC‐40 cells could sensitize CASKI cells to cisplatin. More importantly, the proliferation, migration and invasion of PC11‐PLD1 and PC40‐PLD1 cells with *PLD1* overexpression were significantly improved compared with those of the two types of PLD1 knockout cells. The sensitivity to cisplatin was decreased in PC11‐PLD1 and PC40‐PLD1 cells compared with PC‐11 and PC‐40 cells. In vivo, in the PC‐11 and PC‐40 tumour groups, tumour growth was significantly inhibited and tumour weight (0.95 ± 0.27 g and 0.66 ± 0.43 g vs. 1.59 ± 0.67 g, *p* = 0.0313 and 0.0108) and volume (1069.41 ± 393.84 and 1077.72 mm^3^ ± 815.07 vs. 2142.94 ± 577.37 mm^3^, *p* = 0.0153 and 0.0128) were significantly reduced compared to those in the WT‐CASKI group. Tumour differentiation of the PC‐11 and PC40 cells was significantly better than that of the WT‐CASKI cells. The immunohistochemistry results confirmed that the expression of H‐Ras and p‐Erk1/2 was decreased in PC‐11 and PC‐40 tumour tissues compared with WT‐CASKI tumour tissues. PLD1 promotes CC progression by activating the RAS pathway. Inhibition of PLD1 may serve as an attractive therapeutic modality for CC.

## 1 INTRODUCTION

Cervical cancer (CC) continues to be listed among the top gynaecologic cancers worldwide, ranking fourteenth among all cancers and fourth among women.^1^ Statistics show that the number of deaths associated with CC worldwide in 2018 was 311,000. The total payments for the treatment of cervical uterine and corpus uterine cancers in China were estimated at 11.5 billion RMB in 2015.^2^ Accumulating basic and epidemiological studies have confirmed the key aetiological role of high‐risk human papillomavirus (HPV) infection in the progression of CC. However, viral presence is not sufficient to induce CC,^3^ suggesting that certain molecular events play important roles in its development.

Phospholipase D (PLD), a membrane protein, can hydrolyse phosphatidylcholine to phosphatidic acid (PA) and choline, and PA plays a key role in the regulation of important biological processes.^4^ PLD plays an important role in cytoskeleton formation, cell invasion and migration.^5^ High expression of PLD1 is upregulated in many human malignancies, including liver cancer, breast cancer, prostate cancer, colorectal cancer, multiple myeloma^6^ and CC.^7^ In one of our previous studies,^8^ the proteomic profiles of CC and paracancerous tissue were compared by quantitative proteomics. Through bioinformatics analysis, RAS pathway components, including HRAS, PLD1 and p‐Erk1/2, were found to be aberrantly activated in CC tissue compared with paired normal paracancerous tissues. The immunohistochemistry (IHC) results from the CC tissue microarray (TMA) analysis showed that the increased expression of PLD1 was significantly associated with a tumour size >2 cm and parametrial infiltration. Increased expression of PLD1 and p‐ERK1/2 was adversely related to relapse.^8^ These data suggested that PLD1 may have a potential role in promoting CC progression.

CRISPR/Cas9 has profoundly boosted the progress of genome engineering and has been applied to develop genetically modified animal models and gene therapy.^9^ The stability and reliability of the CRISPR/Cas9 system make it possible to discover new biomarkers and explore new oncogenes in tumour progression.^10^ In this study, the CRISPR/Cas9 technique was used to knock out PLD1 expression in the human CC cell line CASKI. Cell function assays were performed to evaluate the effect of PLD1 on the biological function of CASKI cells in vivo and in vitro. A rescue experiment by *PLD1* overexpression in these knockout cells was performed to further confirm its function. Differences in the expression of RAS pathway components were also detected.

## 2 MATERIALS AND CELL CULTURE

The human CC cell line CASKI, purchased from the Institute of Basic Medicine, Chinese Academy of Medical Sciences & Basic College of Peking Union Medical College, was cultured in RPMI‐1640 medium supplemented with 10% foetal bovine serum and antibiotics (100 U/mL penicillin and 100 μg/mL streptomycin) at 37°C in a humidified atmosphere in a 5% CO_2_ incubator. FuGENE® HD reagents were used for cell transfection according to the manufacturer's instructions. A vector plasmid (GV392) carrying the puromycin resistance gene was used to construct the PLD1‐knockout‐single‐guide RNA (sgRNA) plasmid. The plasmid encoding the PLD1 gene inserted into the pCMV3‐untagged vector and the pCMV3‐untagged negative control vector were purchased from SinoBiologo Chemical Company. The main materials used in this study are shown in Tables S1 and S2.

### 2.1 Use of the CRISPR/Cas9 system to knock out the PLD1 gene in CASKI cells

First, the transfection conditions for the monoclonal cell culture assay and puromycin drug resistance assay were optimized to acquire the optimum transfection efficiency. Next, the selected DNA target sequences (exons 2, 3, 4 and 5 in the case of *PLD1*) were pasted into a CRISPR design tool (http://tools.genome‐engineering.org). The resulting potential target sites with a high‐efficiency score were used to design the sgRNA constructs (20 nucleotides). The nontargeting control sgRNA sequence was CGCTTCCGCGGCCCGTTCAA. Respective sequences were ligated into the expression plasmid LV‐sgCas9‐P2A‐puro (cat. No: GV392) using BbsI (Thermo Fisher), and the details of the sgRNA sequences and plasmids are shown in Table S3. To acquire genome‐edited cell lines, CASKI cells were transfected with mixtures of the respective CRISPR plasmids with puromycin resistance. The negative control CASKI cells were transfected with a nontargeting sgRNA plasmid. Then, the transfected cells were placed in conditioned medium containing 800 ng/mL puromycin for 48–72 h until all the cells in the control group died. Single cells were placed in 96‐well plates and cultured in conditioned medium until they reached a visible size. Single‐cell colonies were selected and expanded. Then, two‐thirds of the harvested cells were used to extract the genomic nucleic acids by a standard phenol/chloroform precipitation procedure. The remaining cells were incubated for further analysis. Isolated genomic DNA was used as a template in PCRs using QuickExtract DNA Extraction Solution according to the manufacturers' instructions (TIANGEN BIOTECH). PCR products (∼523 bp) were electrophoresed on 2% agarose gels, and the samples of interest were purified with the TIANgel Midi Purification Kit as recommended by the manufacturer (TIANGEN BIOTECH). Finally, the DNA fragments were sequenced by TA cloning technology supplied by the GENECHEM company. The paired sequenced primers are presented in Table S3. Clones were examined for frameshift mutations and monoallelic or biallelic deletions/insertions. Upon expansion and growth to confluence in 6 cm dishes, satisfactory cells were trypsinized, pelleted and lysed by adding 100 μl of lysis buffer. Real‐time PCR and Western blot (WB) analysis were used to validate the knockout efficiency of PLD1.

### 2.2 The in vitro effect of PLD1 knockout on the biological functions of CASKI cells

To determine the effect of PLD1 on CASKI cells, the following experimental procedures were performed with WT‐CASKI, PC‐11, PC‐40, PC11‐PLD1 and PC40‐PLD1 cells. A Cell Counting Kit‐8 (CCK‐8) assay was used to detect cell proliferation ability. Transwell Matrigel invasion/migration assays and wound healing assays were used to evaluate the invasion and migration abilities of cells. A colony formation assay was used to detect the colony formation ability of the cells. The epithelial‐mesenchymal transition (EMT)‐related proteins E‐cadherin and vimentin were determined by WB and immunocytochemistry (ICC) assays. Cell viability after cisplatin treatment was determined, and the half‐maximal inhibitory concentration (IC_50_) was calculated by GraphPad Prism 8 software. The gradient concentrations of cisplatin were 100, 50, 25, 12.5, 6.25, 3.125, 1.5625, 0.78125 and 0.3905 μg/mL. ImageJ software was used to calculate the area of wound healing and count the number of colonies formed and migratory and invasive cells. All the experiments were repeated three times.

### 2.3 Overexpression of PLD1 in PLD1 knockout cells

The plasmid encoding the PLD1 gene was inserted into the pCMV3‐untagged vector with the hygromycin gene (Sino Biological Company). The pCMV3‐untagged negative control vector served as a negative control. PC‐11 and PC‐40 cells were then transfected with the PLD1 plasmid or negative control plasmid using Lipofectamine 3000. Real‐time PCR and WB analysis were used to validate the transfection efficiency of PLD1 at the protein and mRNA levels, respectively. An independent sample *t* test was used for statistical evaluation. Primers for real‐time PCR are shown in Table S3.

### 2.4 The in vitro effect of PLD1 overexpression on the biological functions of CASKI cells

The experimental operation is the same as that described in Section 2.3.

### 2.5 Xenograft experiments

PLD1 knockout CASKI cells and WT‐CASKI cells (appropriately 5 × 10^6^ cells) were injected subcutaneously into the left dorsal flank of nude mice (female BALB/c, 5 weeks of age, 6 mice per group). The growth of subcutaneous xenografts in nude mice was observed every 7 days, and the tumour length and width were recorded. The tumour volume was calculated with the following formula: volume = (L × W × W)/2 (where L represents the long diameter and W represents the short diameter). Eight weeks later, all the nude mice were killed by cervical dislocation, and the tumours were dissected and weighed. The tumours were subjected to haematoxylin and eosin (HE) staining, and immunohistochemical staining was carried out to assess the PLD1, H‐Ras, p‐Erk1/2, E‐cadherin and vimentin proteins.

All the procedures performed were approved by the Animal Research Ethics Committee of Capital Medical University.

### 2.6 Statistical analysis

All results are presented as the mean ± standard deviation (SD) of three independent experiments. Statistical analyses were performed using SPSS statistical software (Version 23.0; SPSS, Inc.). Two‐tailed unpaired Student's *t* tests were used to analyse differences between the two groups. For multiple comparisons, the results were corrected with the Bonferroni method. *p* < 0.05 was considered to be statistically significant (*p < 0.05, **p < 0.01, ***p < 0.001, ****p < 0.0001).

## 3 RESULTS

### 3.1 Construction of PLD1‐deficient CASKI cells using the CRISPR/Cas9‐mediated genome editing system

**FIGURE 1:**
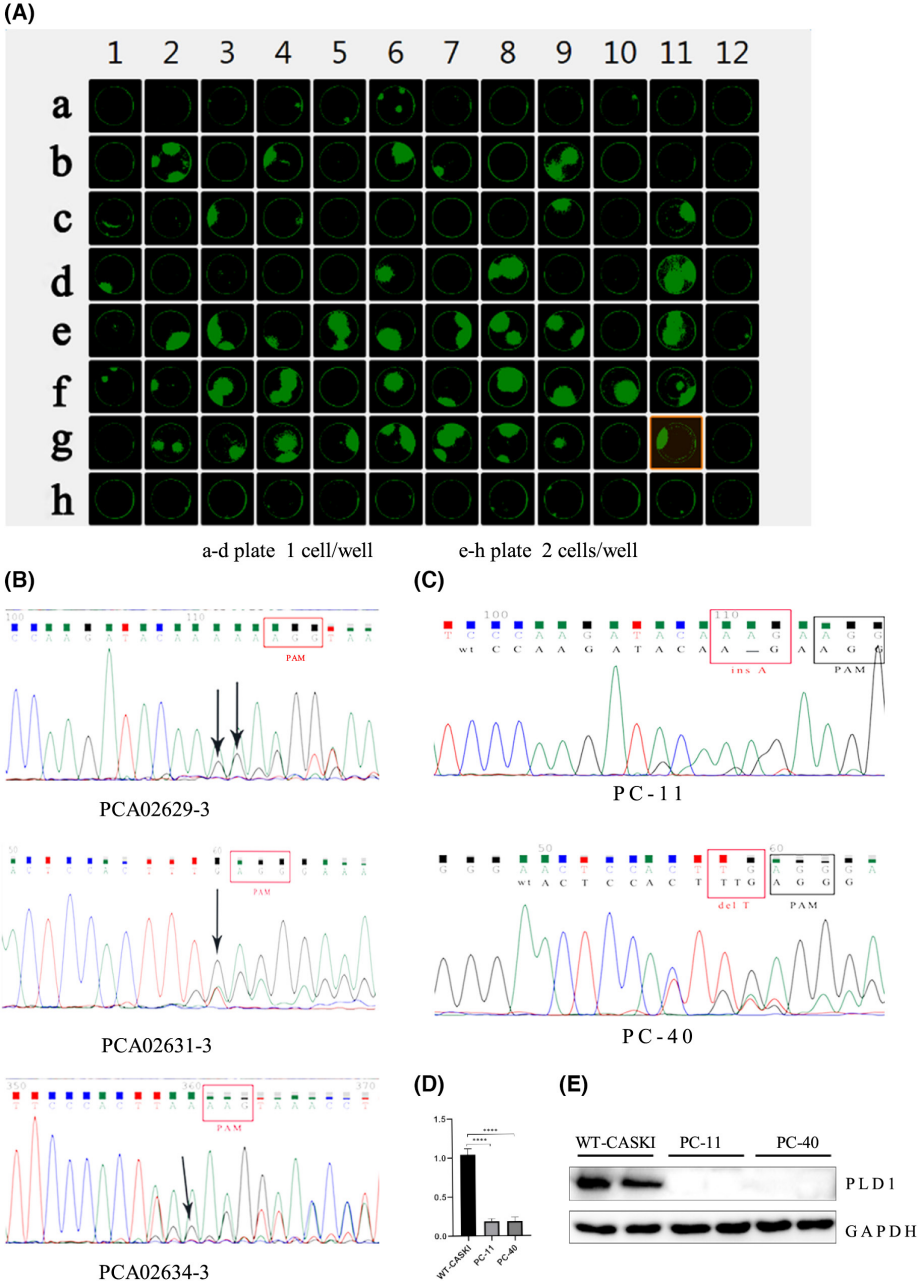
The efficiency of PLD1 knockout by CRISPR/Cas 9. (A) The optimal inoculum density was 2 cells/well for monoclonal culture. (B) Validation of sgRNA cleavage activity through sequence mapping. The three sgRNAs, PCA02629‐3, PCA02631‐3 and PCA02634‐3, showed higher cleavage efficiency. (C) Validation of PLD1 gene mutation in positive cells by Sanger sequencing. The PC‐11 cells carry insertion A base mutation and the PC‐40 cells carry deletion T base mutation (partial sequence). (D) qRT–PCR was used to verify the PLD1 mRNA level (E) Western blot analysis confirmed that the expression of PLD1 was significantly reduced in PC‐11 and PC‐40 cells

Stable PLD1‐knockout CASKI cells and negative control CASKI cells were established by CRISPR/Cas9‐mediated genome editing. Based on the CASKI monoclonal formation assay, the optimal inoculation density of CASKI cells was 2 cells/well, which had a strong monoclonal formation ability (Figure 1A). The optimum puromycin concentration was 800 ng/mL. Six sgRNAs targeting the PLD1 exon 2, 3, 4 and 5 regions were designed to induce PLD1 inactivation. Then, these sgRNAs were cloned into the LV‐sgCas9‐P2A‐puro vector and transfected into CASKI cells. Based on Sanger sequencing maps (Figure 1B), the three most effective plasmids constructed with sgRNAs (PCA02629‐3, PCA02631‐3 and PCA02634‐3) showed higher cleavage efficiency for PLD1 and were transfected into CASKI cells, which were then exposed to puromycin. At the same time, the negative control plasmid with nontargeting PLD1 sgRNA was transfected into CASKI cells, which were then exposed to puromycin.

Some puromycin‐resistant clones were isolated, and Sanger sequencing was performed to confirm the presence of the desired gene mutation. Based on the sequencing results (Figure 1C), #11 monoclonal cells with the PCA02629‐3 plasmid exhibiting the ins1/del4 mutation and #40 monoclonal cells with the PCA02631‐3 plasmid with the del1/del2/ins1 mutation were selected for further study. Two types of PLD1‐knockout CASKI cells (named PC‐11 and PC‐40 cells) and negative control CASKI cells (named WT‐CASKI cells) were selected for further study. The qRT–PCR results showed that PLD1 mRNA levels in PC‐11 and PC‐40 cells were significantly lower than those in WT‐CASKI cells (all *p* < 0.0001; Figure 1D). The WB results also indicated that the expression level of PLD1 was significantly decreased in PC‐11 and PC‐40 cells compared with WT‐CASKI cells (Figure 1E).

### 3.2 The effects of PLD1 on the biological characteristics of CASKI cells

**FIGURE 2:**
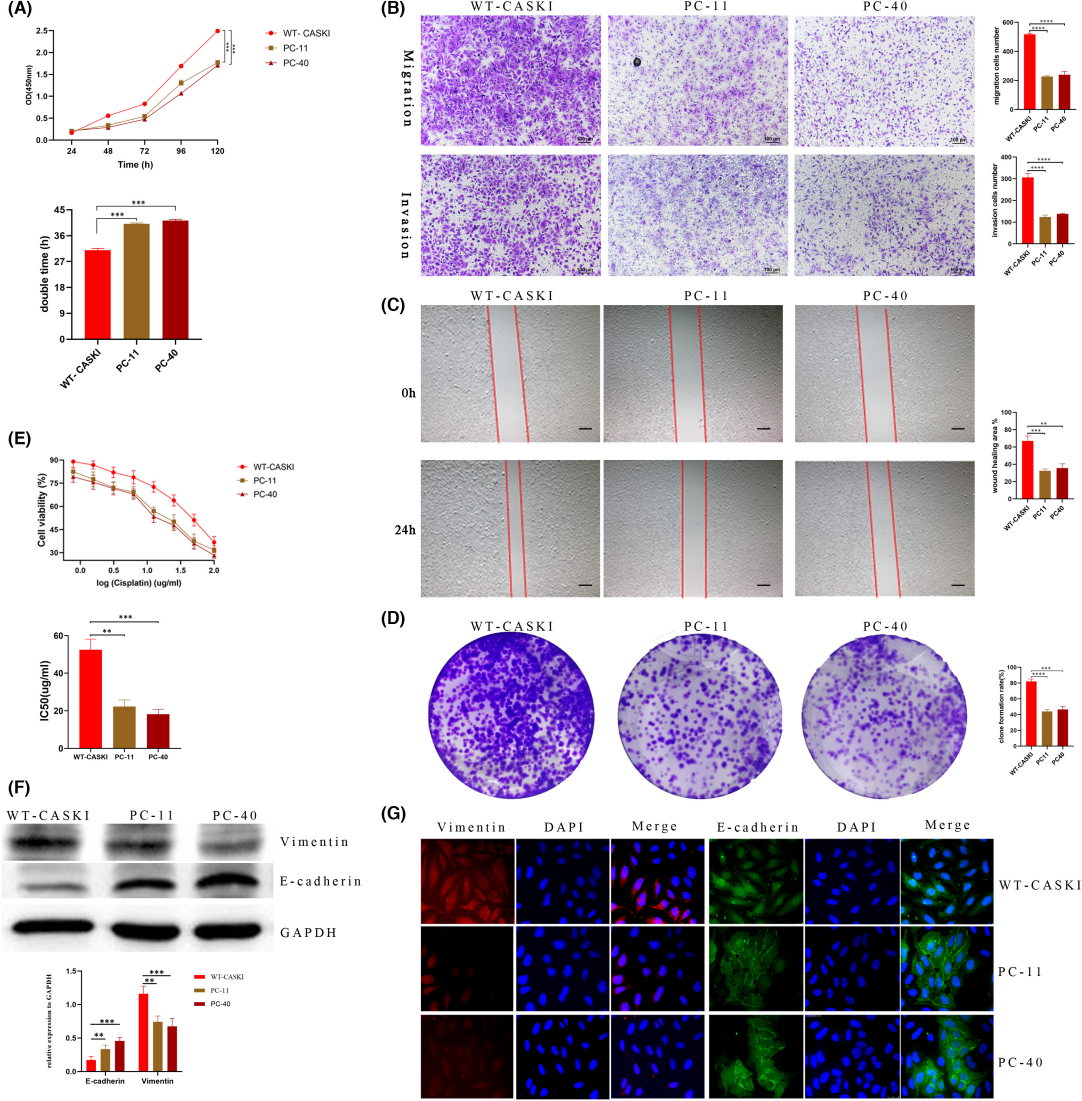
Effects of PLD1 on the biological characteristics of CASKI cells. (A) The CCK‐8 assay growth curves showed that the proliferation activity of PC‐11 and PC‐40 cells was significantly inhibited in comparison to that of WT‐CASKI cells. (B) In the Matrigel migration/invasion assays, PC‐11 and PC‐40 cells demonstrated a significantly weaker ability to migrate and invade than WT‐CASKI cells (both *p* < 0.001). (C) For the scratch assays, PC‐11 and PC‐40 cells had significantly lower migration area percentages than WT‐CASKI cells (32.55 ± 1.68% and 35.62 ± 4.02% vs. 67.01 ± 4.69%, both *p* < 0.001) (D) Colony formation assays showed that the colony formation rate of PC‐11 and PC‐40 cells were both significantly reduced (43.93 ± 2.11% and 46.47 ± 3.28% vs. 82.00 ± 2.54%; all *p* < 0.01, respectively). In addition, the colonies formed by PC‐11 and PC‐40 cells were thinner than those formed by WT‐CASKI cells. (E) PC‐11 and PC‐40 cells exhibited higher sensitivity to cisplatin and the IC_50_ values significantly decreased in comparison with WT‐CASKI cells (22.25 ± 3.52 μg/mL and 18.12 ± 2.72 μg/mL vs. 52.45 ± 5.67 μg/mL, *p* = 0.0014 and 0.0007, respectively). (F, G) Western blot and immunocytochemistry results showed that vimentin was poorly expressed, whereas E‐cadherin was highly expressed in PC‐11 and PC‐40 cells. *p < 0.05, **p < 0.01, ***p < 0.001, ****p < 0.0001

The CCK‐8 assay growth curves showed that the proliferation activity of PC‐11 and PC‐40 cells was significantly inhibited compared with that of WT‐CASKI cells. The doubling time of PC‐11 cells and PC‐40 cells was significantly prolonged compared with that of WT‐CASKI cells (40.11 ± 0.28 h, 41.27 ± 0.40 h vs. 30.97 ± 0.57 h, both *p* < 0.01; Figure 2A). In the Matrigel migration/invasion assay, the invasion and migration abilities of PC‐11 and PC‐40 cells were significantly reduced compared with those of WT‐CASKI cells (all *p* < 0.01; Figure 2B). For the scratch assays, PC‐11 and PC‐40 cells had significantly lower migration area percentages than WT‐CASKI cells (32.55 ± 1.68% and 35.62 ± 4.02% vs. 67.01 ± 4.69%, both *p* < 0.001; Figure 2C). Colony formation assays showed that the colony formation rate of PC‐11 and PC‐40 cells were both significantly reduced compared with WT‐CASKI (43.93 ± 2.11% and 46.47 ± 3.28% vs. 82.00 ± 2.54%; all *p* < 0.01; Figure 2D). In addition, the clones formed by PC‐11 and PC‐40 cells were thinner than those formed by WT‐CASKI cells (Figure 2D). PC‐11 and PC‐40 cells exhibited high sensitivity to cisplatin, and the half‐maximal inhibitory concentration (IC50) values significantly decreased in comparison with WT‐CASKI cells (22.25 ± 3.52 μg/mL and 18.12 ± 2.72 μg/mL vs. 52.45 ± 5.67 μg/mL, *p* = 0.0014 and 0.0007, respectively; Figure 2E). Furthermore, in contrast to WT‐CASKI cells, vimentin was poorly expressed, whereas E‐cadherin was highly expressed in PC‐11 and PC‐40 cells, indicating that knockout of PLD1 could suppress the EMT process of CASKI cells (Figure 2F,G).

### 3.3 PLD1 rescue in the knockout cell lines by PLD1 overexpression

**FIGURE 3:**
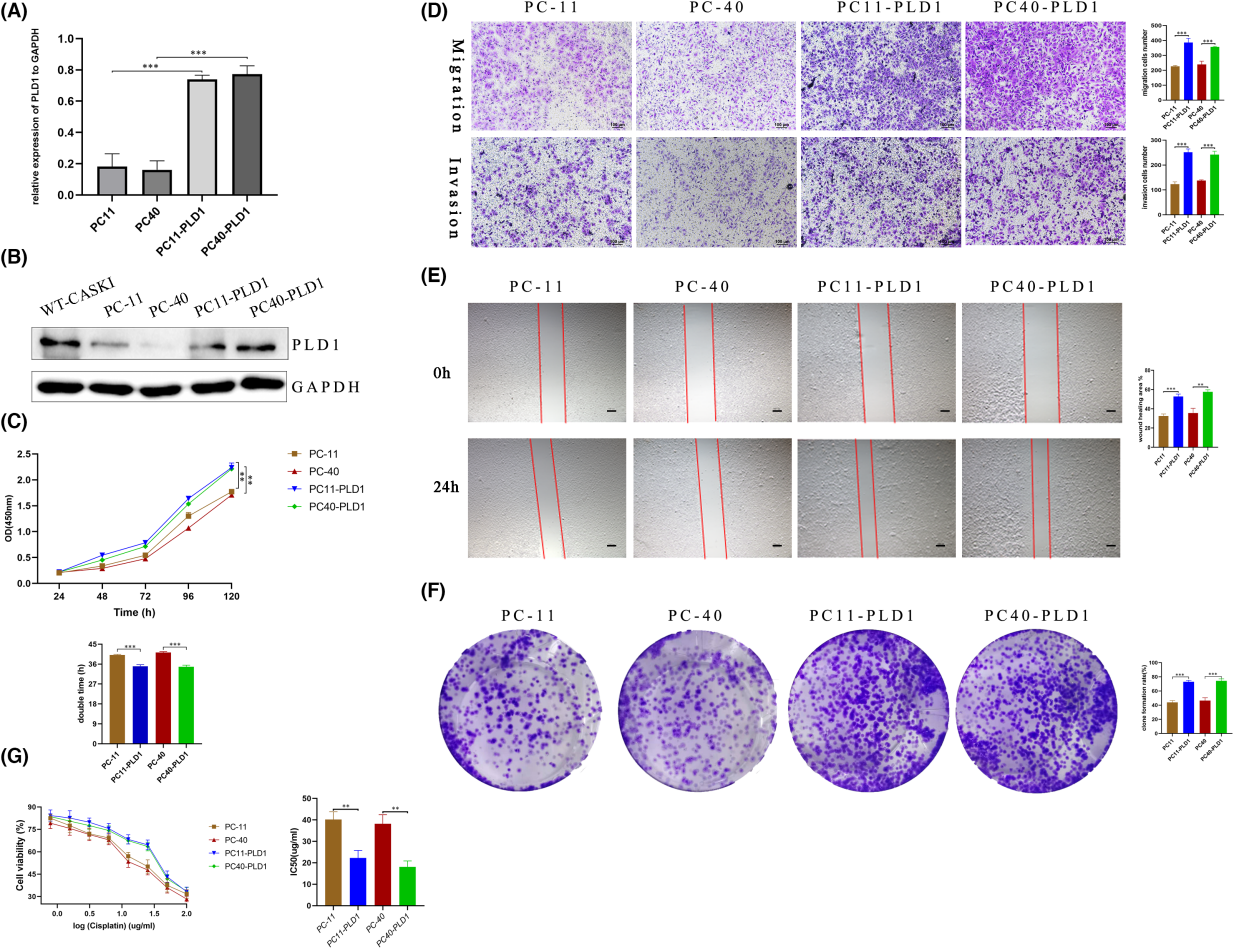
Effects of PLD1 overexpression on the biological characteristics of CASKI cells. (A) qRT–PCR was used to verify the PLD1 mRNA level. (B) Western blot analysis confirmed that the expression of PLD1 was significantly increased in PC11‐PLD1 and PD40‐PLD1 cells. (C) The CCK‐8 assay growth curves suggested that PC11‐PLD1 and PC40‐PLD1 cells proliferated faster than PC11 and PC‐40 cells, and the doubling time of PC11‐PLD1 and PC40‐PLD1 cells was significantly shortened in comparison to that of PC‐11 and PC‐40 cells (35.05 ± 0.68 h vs. 40.11 ± 0.28 h, 34.78 ± 0.76 h vs. 41.27 ± 0.40 h, respectively, all *p* < 0.05). (D) Matrigel migration/invasion assay results showed that PC11‐PLD1 and PC40‐PLD1 cells showed a higher ability to invade and migrate than PC‐11 and PC‐40 cells. (E) The scratch assay results showed that PC11‐PLD1 and PC40‐PLD1 cells had significantly higher migration area percentages than PC‐11 and PC‐40 cells (52.85 ± 2.0% vs. 32.55 ± 1.70%, 57.54 ± 1.80% vs. 35.62% ± 4.02%, all *p* < 0.001). (F) Colony formation assays showed that the colony formation rate of PC11‐PLD1 and PC40‐PLD1 cells was significantly greater than that formed by the two PLD1 knockout cells (43.93 ± 2.11% vs. 73 ± 1.72%, 46.47 ± 3.28% vs. 74.4 ± 2.27%, all *p* < 0.01). (G) PC11‐PLD1 and PC40‐PLD1 showed lower sensitivity to cisplatin, and the IC_50_ values significantly increased compared with those of PC‐11 and PC‐40 cells (40.20 ± 3.59 μg/mL vs. 22.25 ± 3.52 μg/mL, 38.15 ± 4.26 μg/mL vs. 18.12 ± 2.72 μg/mL, *p* = 0.0035 and 0.0024, respectively)

In the PLD1 rescue experiment, PC‐11 and PC‐40 cells were transfected with the PLD1‐overexpression plasmid or the pCMV3‐untagged negative control (named PC11‐PLD1 and PC40‐PLD1 cells). The qRT–PCR results showed that PLD1 mRNA levels in PC11‐PLD1 and PC40‐PLD1 cells were significantly higher than those in PC‐11 and PC‐40 cells (all *p* < 0.01; Figure 3A). The WB analysis results revealed that the expression of PLD1 was significantly increased in PC11‐PLD1 and PC40‐PLD1 cells compared with PC‐11 and PC‐40 cells (Figure 3B). Compared with WT‐CASKI cells (transfected with the pCMV3‐untagged negative control plasmid), the PLD1 expression rescue efficiency reached average of 71.33% and 74.54% in PC11‐PLD1 and PC40‐PLD1 cells, respectively.

### 3.4 The effect of PLD1 overexpression on the biological characteristics of CASKI cells

In contrast, the CCK‐8 assay growth curves showed that the doubling time of PC11‐PLD1 and PC40‐PLD1 cells was significantly shorter than that of PC‐11 and PC‐40 cells (35.05 ± 0.68 h vs. 40.11 ± 0.28 h, 34.78 ± 0.76 h vs. 41.27 ± 0.40 h, respectively, all *p* < 0.05; Figure 3C). In the Matrigel invasion/migration assay, PC11‐PLD1 and PC40‐PLD1 cells demonstrated a greater ability to invade and migrate (all *p* < 0.001, Figure 3D) through the membrane than the two types of PLD1 knockout cells. The scratch assay results showed that the migration area percentage of PC11‐PLD1 and PC40‐PLD cells was larger than that of PC‐11 and PC‐40 cells (52.85 ± 2.0% vs. 32.55 ± 1.70%, 57.54 ± 1.80% vs. 35.62% ± 4.02%, all *p* < 0.001, Figure 3E). Colony formation assays showed that the colony formation rate of PC11‐PLD1 and PC40‐PLD1 cells was significantly greater than that formed by the two PLD1 knockout cells (43.93 ± 2.11% vs. 73 ± 1.72%, 46.47 ± 3.28% vs. 74.4 ± 2.27%, all *p* < 0.01, Figure 3F). PC11‐PLD1 and PC40‐PLD1 cells exhibited lower sensitivity to cisplatin, and the IC_50_ values significantly increased in comparison with PC‐11 and PC‐40 cells (40.20 ± 3.59 μg/mL vs. 22.25 ± 3.52 μg/mL, 38.15 ± 4.26 μg/mL vs. 18.12 ± 2.72 μg/mL, *p* = 0.0035 and 0.0024, respectively; Figure 3G).

### 3.5 PLD1 knockout inhibits tumour growth in vivo

**FIGURE 4:**
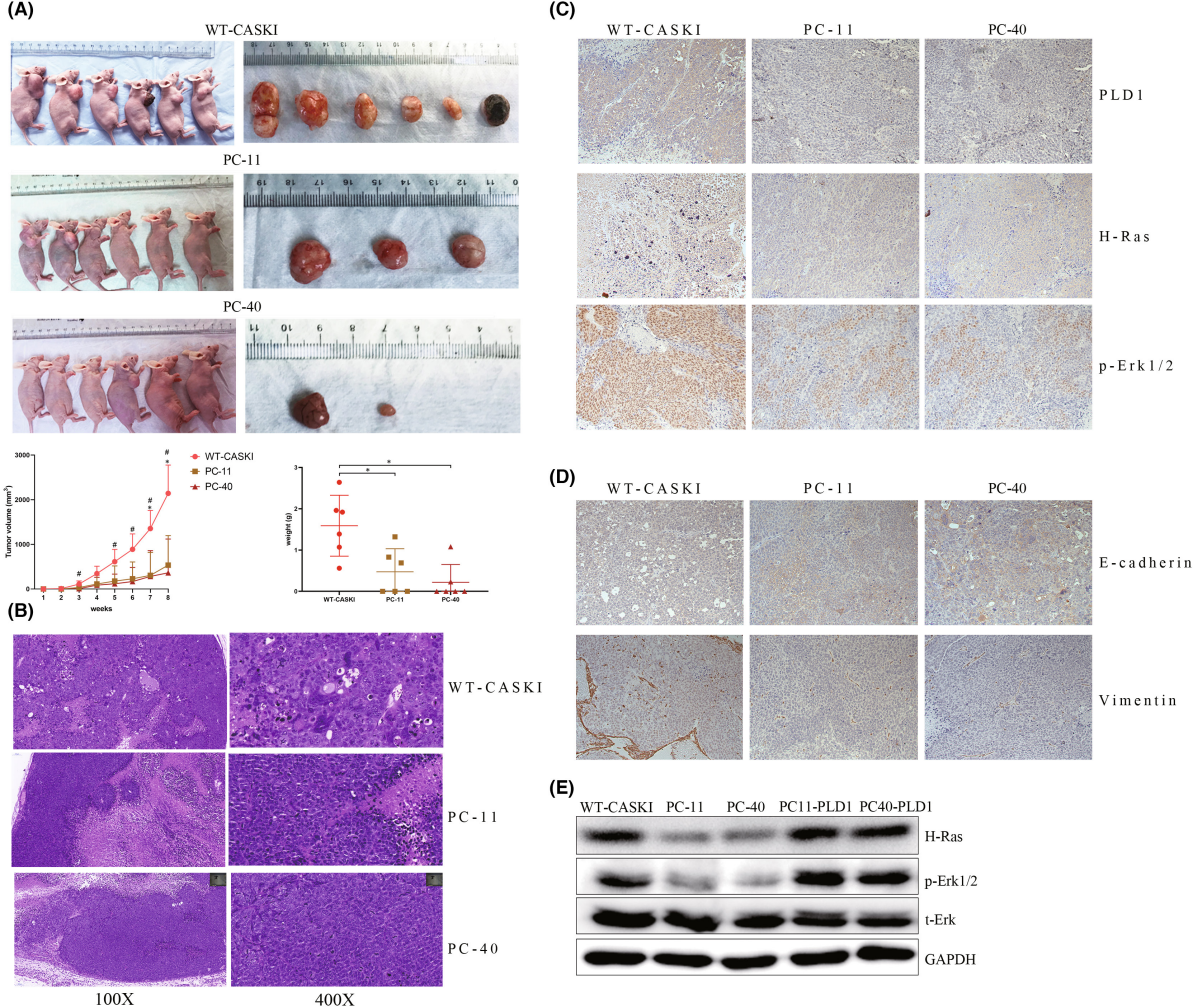
PLD1 knockout inhibits tumour growth in vivo. (A) PC‐11 and PC‐40 cells had smaller tumours than WT‐CASKI cells, and the nude mouse tumorigenesis rate was lower than that in the WT‐CASKI group (50% and 33.3% vs. 100%). Tumour weight. Data are presented as the mean ± SD. Growth curves of tumour volumes. (B) HE results showed that tumour differentiation of PC‐11 and PC‐40 cells was significantly better than that of WT‐CASKI cells. (C) The representative images of p‐ERK1/2 and H‐Ras immunohistochemical staining in tumour tissues. (D) The representative images of E‐cadherin and vimentin immunohistochemical staining in tumour tissues. (E) The WB results showed that the expression of p‐ERK1/2 and H‐Ras was significantly decreased in PC‐11 and PC‐40 cells compared with WT‐CASKI cells (*p* = 0.0066 and 0.0031, *p* = 0.0005 and 0.0007, respectively). In PC11‐PLD1 and PC40‐PLD1 cells, the expression of p‐ERK1/2 and H‐Ras was significantly increased compared with that in PC‐11 and PC‐40 cells (all *p* < 0.05). *p < 0.05, **p < 0.01, ***p < 0.001, #*p* < 0.05, ##*p* < 0.01, ###p < 0.001

After subcutaneous injection, PC‐11 and PC‐40 tumours were observed much later than WT‐CASKI tumours (14th and 21st days vs. 7th day). At the end of the study, in the PC‐11 and PC‐40 groups, the nude mouse tumorigenesis rate was lower than that in the WT‐CASKI group (50% and 33.3% vs. 100%). In the PC‐11 and PC‐40 groups, tumour growth was significantly inhibited, and tumour weight (0.95 ± 0.27 and 0.66 ± 0.43 vs. 1.59 ± 0.67 g; *p* = 0.0313 and 0.0108) and volume (1069.41 ± 393.84 and 1077.72 ± 815.07 vs. 2142.94 ± 577.37 mm^3^; *p* = 0.0153 and 0.0128) were significantly reduced compared with those in the WT‐CASKI group (Figure 4A). The HE results showed that PC‐11 and PC‐40 WT‐CASKI tumours were both typical nonkeratinizing squamous cell carcinomas, but the differentiation of PC‐11 and PC‐40 tumours was significantly better than that of WT‐CASKI tumours (Figure 4B). The immunohistochemistry results showed that the expression of both H‐Ras and p‐Erk1/2 was significantly decreased in PC‐11 and PC‐40 tumour tissues compared with WT‐CASKI tumour tissues (Figure 4C). Additionally, the immunohistochemistry results showed that PLD1 knockout improved the expression of E‐cadherin and inhibited the expression of vimentin in tumour tissues, indicating that PLD1 knockout inhibited the EMT process of CASKI cells in vivo (Figure 4D).

### 3.6 Correlation between PLD1 expression and the Ras pathway

The WB results showed that the expression of p‐ERK1/2 and H‐Ras was significantly decreased in PC‐11 and PC‐40 cells compared with WT‐CASKI cells (*p* = 0.0066 and 0.0031, *p* = 0.0005 and 0.0007, respectively; Figure 4E). However, in PC11‐PLD1 and PC40‐PLD1 cells, the expression of p‐ERK1/2 and H‐Ras was significantly increased compared with that in PC‐11 and PC‐40 cells (all *p* < 0.05, Figure 4E).

## 4 DISCUSSION

PLD1 can hydrolyse major membrane glycerophospholipids to the lipid second messenger PA, which plays a role in disease processes such as cancer.^11^ PLD1 and its product, PA, participate in various physiological and pathological processes, such as cell growth, invasion, metabolism and autophagy.^10^, ^12^, ^13^ PLD1 exhibits a higher expression level in colorectal tumours, bladder cancer and breast cancer.^14^, ^15^, ^16^ The high expression of PLD1 in tumour tissues is related to a poor prognosis in patients with colorectal cancer and bladder cancer.^15^, ^16^ However, there are few reports on PLD1 in CC. In our previous study, liquid chromatography with tandem mass spectrometry was used to compare the proteomics between CC and paired paracancerous tissues. Bioinformatics analysis showed that the expression of PLD1, H‐Ras and p‐ERK1/2 in CC tissues was significantly higher than that in paired paracancerous tissues. The IHC results of the CC TMA demonstrated that the expression levels of PLD1, H‐RAS and p‐ERK1/2 in CC tissues were significantly higher than those in paired paracancerous tissues.^8^ In addition, the elevated expression level of PLD1 was significantly related to a tumour size >2 cm and parametrial invasion. Moreover, the increased expression levels of PLD1 and p‐ERK1/2 were adversely related to patient relapse and survival.^8^ PLD1 has a potential role in promoting CC tumorigenesis.

CRISPR/Cas9 has been widely applied to the discovery of tumour‐driven genes and tumour therapies.^17^ Because it can induce chromosomal rearrangements, such as insertions, deletions and translocations, efficiently and accurately and generate targeted breaks at desired regions in a genome, the CRISPR/Cas9 system has profoundly promoted the progress of genome engineering.^18^, ^19^ Co‐expression of Cas9 with sgRNAs has been used to construct a variety of cell and animal models for the study of tumour progression and is regarded as an option for reducing and identifying tumour target genes in tumours.^9^, ^20^ In this study, the PLD1 gene in CASKI cells was knocked out by CRISPR/Cas9 genome editing technology. Two stable PLD1‐knockout cell lines, PC‐11 and PC‐40 cells, were established that exhibited ins1/del4 mutations and del1/del2/ins1 mutations, respectively. In this way, the potential role of PLD1 in the progression of CC could be reliably explored.

In the present study, PC‐11 and PC‐40 cells exhibited dramatic decreases in proliferation, migration and invasion. Sensitivity to cisplatin was also reversed. In contrast, when the expression of PLD1 was rescued in PC‐11 and PC‐40 cells, the proliferation, migration and invasion abilities of PC11‐PLD1 and PC40‐PLD1 were improved. Thus, PLD1 could affect the biological characteristics of CASKI cells, as confirmed by PLD1 knockout and PLD1 rescue experiments. Similarly, inhibition of *PLD1* suppresses cancer cell biological functions in prostate cancer, hepatocellular carcinoma and bladder cancer.^15^, ^21^, ^22^ In addition, the tumorigenesis ability of CASKI cells in nude mice was also inhibited in this manner. Xiao and colleagues^21^ also demonstrated that PLD1 inhibitors could decrease the biological activity of cells of the human liver cancer cell line HepG2 in vivo and in vitro. EMT, whereby cells can acquire more migratory and invasive abilities, is attributed to tumour metastasis. Decreased expression of vimentin and increased expression of E‐cadherin are the key markers of EMT.^23^ In this study, the expression of vimentin was significantly decreased and that of E‐cadherin was increased in PC‐11 and PC‐40 cells, indicating that knockout of PLD1 could attenuate EMT progression in CASKI cells, and this phenomenon was observed in mouse tumour tissues in vivo. Previous studies demonstrated that inhibition of PLD1 could attenuate tumour EMT in hepatocellular carcinoma mice,^21^ and PLD1 mediated EMT in hepatocellular carcinoma.^24^

The RAS/RAF/MEK/ERK (MAPK) signalling pathway is pivotal in the cell signalling cascade that regulates cell survival, growth, migration and invasion.^25^ PLD1 is a critical downstream mediator of HRAS‐induced tumour formation. PLD1 plays a key role in the phosphorylation of ERK, and its biological function is regulated by RAS/RAL genes.^26^ PLD1 has been shown to facilitate Ras and ERK activation via interaction with PEA‐15.^27^ In this analysis, the expression of H‐Ras and p‐Erk1/2 was significantly reduced in PC‐11 and PC‐40 cells. In xenograft experiments, the IHC results showed that the expression of H‐Ras and p‐Erk1/2 was significantly decreased in PC‐11 and PC‐40 tumour tissues. These data suggested that PLD1 promoted CC progression by activating the Ras pathway.

## 5 CONCLUSIONS

PLD1 was activated in CC tissues and cells. PLD1 knockout inhibited the biological activity of CASKI cells in vitro and in vivo. PLD1 possibly promoted CC progression by activating the Ras pathway and phosphorylating ERK1/2. *PLD1* knockout may be potentially effective for treating CC.

## AUTHOR CONTRIBUTIONS

**Meiying Song:** Conceptualization (lead); data curation (lead); formal analysis (lead); funding acquisition (equal); investigation (equal); methodology (lead); project administration (lead); resources (lead); software (lead); supervision (lead); validation (lead); visualization (lead); writing – original draft (equal); writing – review and editing (equal). **qianlong Meng:** Conceptualization (lead); data curation (lead); formal analysis (lead); funding acquisition (equal); investigation (lead); methodology (lead); project administration (lead); resources (lead); software (lead); supervision (equal); validation (lead); visualization (lead); writing – original draft (lead); writing – review and editing (lead). **Xuan Jiang:** Conceptualization (equal); data curation (equal); formal analysis (equal); funding acquisition (equal); investigation (equal); methodology (equal); project administration (equal); resources (equal); software (equal); supervision (equal); validation (equal); visualization (equal); writing – original draft (equal); writing – review and editing (equal). **Meizhu Xiao:** Conceptualization (equal); data curation (equal); formal analysis (equal); investigation (equal); methodology (equal); project administration (equal); resources (equal); software (equal); supervision (equal); validation (equal); visualization (equal); writing – original draft (supporting); writing – review and editing (supporting). **jun liu:** Conceptualization (equal); data curation (equal); formal analysis (equal); funding acquisition (equal); investigation (equal); methodology (equal); project administration (equal); resources (equal); software (equal); supervision (equal); validation (equal); visualization (equal); writing – original draft (equal); writing – review and editing (equal). **zhenyu zhang:** Conceptualization (equal); data curation (equal); formal analysis (equal); funding acquisition (equal); investigation (equal); methodology (equal); project administration (equal); resources (equal); software (equal); supervision (equal); validation (equal); visualization (equal); writing – original draft (equal); writing – review and editing (equal). **jing wang:** Conceptualization (lead); data curation (lead); formal analysis (lead); funding acquisition (equal); investigation (equal); methodology (equal); project administration (lead); resources (lead); software (lead); supervision (lead); validation (equal); visualization (equal); writing – original draft (equal); writing – review and editing (equal). **Huimin Bai:** Conceptualization (lead); data curation (lead); formal analysis (lead); funding acquisition (lead); investigation (lead); methodology (lead); project administration (lead); resources (lead); software (lead); supervision (lead); validation (lead); visualization (lead); writing – original draft (supporting); writing – review and editing (lead).

## CONFLICT OF INTEREST

The authors have no conflicts of interest to declare.

## CONSENT FOR PUBLICATION

All the authors have reviewed the manuscript and the related files and consented to its publication.

## Supporting information


Table S1

Table S2

Table S3

## Data Availability

The data sets supporting the results of this article are included within the article and its additional files.
