# Supplementary material for: Phospholipase D1 promotes cervical cancer progression by activating the RAS pathway
Source: J Cell Mol Med. 2022 Jun 30;26(15):4244–53. doi: 10.1111/jcmm.17439 (PMC9344829; doi:10.1111/jcmm.17439)
Supplement: Supplementary file 1 — Table S1 [file JCMM-26-4244-s001.pdf]

Table S1 The main materials used in this work.

**Materials**	**Supplier**
Human cervical cancer cell line CASKI	Institute of Basic Medicine, Chinese Academy of Medical Sciences &; Basic College of Peking Union Medical College
**Cell culture**	
RPMI-1640	Corning Inc, USA
Fetal bovine serum (FBS)	
Trypsin-EDTA	Sigma-Aldrich Inc, Germany
Penicillin streptomycin	
**CRISPR/Case9 editing system**	
FuGENE® HD	Promega, USA
Primer（R&F）	Shanghai Genechem, China
Oligo primers	Shanghai Generay Biotech, China
TOP10 competent cells	Shanghai Genechem, China
2×Taq Plus Master Mix	Vazyme, China
BsmBI	Thermo Fisher, USA
T4 DNA Ligase	TIANGEN BIOTECH, China
TIANgel Midi Purification Kit	
EndoFree Mini Plasmid Kit II	
Tryptone	OXOID, USA
Yeast Extract	
Agar Powder	
Agarose	Sigma-Aldrich Inc, Germany
DL-Dithiothreitol(DTT)	Sangon Biotech (Shanghai) Co.,Ltd
250bp-II DNA ladder	Shanghai Generay Biotech Co.,Ltd
