# Supplementary material for: Phospholipase D1 promotes cervical cancer progression by activating the RAS pathway
Source: J Cell Mol Med. 2022 Jun 30;26(15):4244–53. doi: 10.1111/jcmm.17439 (PMC9344829; doi:10.1111/jcmm.17439)
Supplement: Supplementary file 2 — Table S2 [file JCMM-26-4244-s003.pdf]

Table S2 Details of primary and secondary antibodies used for western blotting or immunocytochemistry staining.

**Antibodies**	**Catalog No**	**Supplier**
Rabbit anti-human HRAS	ab201054	ABCAM, Inc
Rabbit anti-human PLD 1	ab50695	
Mouse anti-human GAPDH	ab8245	
Rabbit anti-human p-ERK1/2(Thr202/Tyr204)	#4370	Cell Signaling Technology, Inc
Rabbit anti-human E-cadherin	#3195C	
Rabbit anti-human Vimentin	#5741	
