# Supplementary material for: Phospholipase D1 promotes cervical cancer progression by activating the RAS pathway
Source: J Cell Mol Med. 2022 Jun 30;26(15):4244–53. doi: 10.1111/jcmm.17439 (PMC9344829; doi:10.1111/jcmm.17439)
Supplement: Supplementary file 3 — Table S3 [file JCMM-26-4244-s002.pdf]

Table S3

1. The details of respective sgRNAs for PLD1

sgRNA sequence	location	Plasmid	Resistance gene	Vectors
AGCGATCCCAAGATACAAGA	Exon5	PCA02629-3	puromycin	LV-sgCas9-P2A-puro
AATATACAGACGTATCTCTC	Exon5	PCA02630-3	puromycin	LV-sgCas9-P2A-puro
CACGCGGGAACTCCACTTTG	Exon2	PCA02631-3	puromycin	LV-sgCas9-P2A-puro
GAGAGATACGTCTGTATATT	Exon4	PCA02632-3	puromycin	LV-sgCas9-P2A-puro
TACACTATTGAATTAACACA	Exon3	PCA02633-3	puromycin	LV-sgCas9-P2A-puro
GCATCCCCATTCCCACTAGA	Exon2	PCA02634-3	puromycin	LV-sgCas9-P2A-puro

2. Primers for respective plasmids

**Primer**	**Sequence (5' to 3')**	plasmid
PLD1 F1	TGAAAAACGAGCCACGGGTA	pca02629, pca02630, pca02631, pca02632
PLD1 R1	ACGCTCTGACCCTTGTTGTAG	
PLD1 F2	GGAGGAACAGCGTTTGTAGGA	pca02633, pca02634
PLD1 R2	TCCATTCAACTTGTATACTGGCA	

3. PLD1 primer sequence for PLD1 overexpression

PLD1 forward primer	**5'**-CCCAGCGATCCCAAGATACAA-**3'**
PLD1 reverse primer	**5'**-GACAGCCGGAGAGATACGTCT-**3'**
